# Completeness of police reporting of traffic crashes in Nepal: Evaluation using a community crash recording system

**DOI:** 10.1080/15389588.2021.2012766

**Published:** 2022-01-14

**Authors:** Anish Khadka, John Parkin, Paul Pilkington, Sunil Kumar Joshi, Julie Mytton

**Affiliations:** a Nepal Injury Research Centre, Kathmandu Medical College Public Limited, Kathmandu, Nepal; b Centre for Transport and Society, University of the West of England, Bristol, UK; c Centre for Academic Child Health (CACH), University of the West of England, Bristol, UK

**Keywords:** Road traffic crash, road traffic injury, Local Record Keeper, under-reporting, Nepal

## Abstract

**Objective:**

Accurate road crash reporting is essential for evaluating road safety interventions and plans. Under-reporting of road traffic crashes, injuries, and fatalities in police records has been widely described. The aim of this study was to apply and evaluate a community crash recording system, and assess the quality of the data in comparison with traffic police data in Nepal.

**Methods:**

The crash data collection methodology involved recruiting Local Record Keepers working and living at locations known to be at a high risk of crashes. Six shopkeepers were recruited at three crash locations and trained to complete collision incident forms for crashes occurring over one year on the section of road visible from their premises. Manual traffic volume and pedestrian counts, and spot speed surveys were conducted. Data were compared with District police records for the same period and locations.

**Results:**

Over 12 months, 110 crashes were recorded by the Local Record Keepers. Of these, 70 resulted in 145 injuries (5 fatalities, 62 severe, and 78 minor injuries), while 40 resulted only in property damage. Comparable police data recorded 23 crashes, of which 18 crashes resulted in 27 injuries (8 fatalities, 13 serious, and 6 minor injuries), and 5 crashes in property damage only. The difference in recording of fatal and serious injuries was statistically significant (χ^2^(1) = 19.94, p < 0.001). The police reporting rate was highest for fatalities (62.5%) but only 11.6% and 7.1% for property damage cases and minor injuries respectively, and 3.8% for single-vehicle crashes. Compared to the Local Record Keeper data, the overall police crash reporting rate was 19.7%.

**Conclusions:**

Local Record Keepers’ recording of road traffic crashes and casualties is feasible and provides a more complete record than routinely collected police data. The low reporting rate in the police records of minor injury, property damage, and single-vehicle crashes suggest significant underestimation and bias in the reporting of the actual burden of road traffic crashes. Local Record Keeper recording is a viable method for validating police reports.

## Introduction

Nepal has one of the highest estimated road traffic crash (RTC) fatality rates in South Asia, with 15.9 fatalities per 100,000 population (World Health Organization 2019). Based on police reports aggregated from local traffic police offices, there were 10,178 road crashes, with 2,384 fatalities, 4,250 serious injuries, and 8,290 minor injuries in 2016 (Nepal Police 2019). This is about half of the World Health Organization modeled estimate of 4,622 fatalities, suggesting under-reporting. Most existing epidemiological studies in Nepal are dependent on police data (Dhakal 2018; Karkee and Lee 2016). Data inaccuracies lead to under and biased estimation of road traffic injuries (RTIs), and an inability to accurately suggest appropriate preventive measures (Amoros et al. 2006).

Under-reporting in police records is a worldwide issue (Dandona et al. 2008; Muni et al. 2021). Yadav et al. (2021) conducted a systematic review and found the crash reporting rate to be higher in high-income countries (HICs) for fatal injuries (35% to 96.6%) compared to low- and middle-income countries (LMICs, 4.2% to 77.8%). The reporting rate of non-fatal injuries was found to be between 16% and 82% for HICs and 6.7% and 24.7% for LMICs, highlighting a decrease with reduced injury severity levels. The integration of multiple datasets is a common methodology used to draw conclusions about the quality and reliability of police data and obtain a more complete picture of the burden of road traffic injuries and crashes (Rosman 2001; Short and Caulfield 2016). Hospital records are commonly used to ascertain police data quality and completeness (Aptel et al. 1999; Janstrup et al. 2016; Van et al. 2006). Singh et al. (2018) conducted a cross-sectional study in Chandigarh, India, reporting only 15% of hospital records matched police records, while in Malaysia, Kamaluddin et al. (2019) reported a matching rate of only 4.1%. Hospital records are limited in their ability to validate police data as they will only capture injured patients attending healthcare services, and health facilities generally lack injury surveillance systems (Magnus et al. 2020).

Kamaluddin et al. (2018) promote the use of self-reported crash data. However, such data are prone to recall and social desirability biases. Thus, there remains an opportunity to explore alternative methods to record local crash and injury data that can provide a reliable source for validating routine data, intervention evaluation, and policy planning. Community-based surveys have the potential to capture more complete road traffic injury (RTI) data than police or hospital records (McGee et al. 2004). One such approach was developed by Van der Horst et al. (2017) in Bangladesh, where they trained community-based Local Record Keepers (LRK) to maintain a recording system for crashes resulting in injury or fatality for a pre-defined geographic area, and used it to evaluate the impact of road safety interventions. The aim of this study was to apply and evaluate LRK methodology in Nepal for crash data collection, and compare the quality of the data with police records.

## Methods

### Study design

We adapted for the Nepal context the principles of the LRK methodology piloted by Van der Horst et al. (2017) in Bangladesh. The record keepers were trained how to complete the collision incident form, the definition of a collision, and instructed on the boundaries of the selected stretch of road in which they were to record crashes. Adaptations included: revising the data collection form to account for the difference in vehicle categories between the two countries, and crash type recorded (both injury and non-injury crashes were recorded in Nepal, while only injury crashes were recorded in Bangladesh). Retrospective road traffic collision and casualty data were extracted from traffic police records for comparison purposes, and contextual traffic and pedestrian count and traffic speed surveys were undertaken.

### Place and time of study

The East-West highway (Asian Highway 2, AH2) is a two-lane single carriageway road with a mix of diverse vehicle types. The highway caters for a significant part of Nepal’s freight and passenger traffic, and links south to India. Three lengths of highway near Hetauda, a sub-metropolitan city in the Makwanpur district of Bagmati province were selected (Pashupatinagar, Nawalpur, and Basamadi). Maps and photographs are shown in Appendices Figures A1, A2, A3, and A4 (see online supplement). The sites were selected as being at high risk of crashes based on the analysis of traffic police records for the year 1 April 2017 to 31 March 2018, site visits and community engagement activities. All three locations encompass ribbon settlements with traffic generators including schools, industrial sites, and markets. The lengths of the highway were shorter than the equivalent police reporting areas at the same locations by up to 1.4 km. Historically, the police records did not state a precise crash location. Working with the local police, more accurate records of crash locations within these locations were recorded for the duration of the study.

### Data collection

#### Local Record Keeper data

Community engagement suggested shopkeepers would make appropriate LRKs. Personal contact was made with a number of shopkeepers working in the selected study sites. Shopkeepers were informed about the proposed study and asked if they were interested to learn more. Through a series of discussions, we recruited six LRKs to collect data for a year from 1 May 2019 to 30 April 2020. LRKs met the following criteria: a) motivated to reduce RTCs and their community impact; b) having a shop location overlooking the highway; c) being resident at the location; d) having family members able to assist; and e) being able to read and write. Using a data collection form (Appendix Figure A5, online supplement), LRKs recorded crashes they witnessed, or about which they were informed. They were encouraged to engage family and community members in the activity. Each of the three lengths of the highway was divided into two sub-sections, one per LRK. They recorded crashes within the field of view of their shop.

The LRKs recorded the date, time and location of road traffic collisions, the number and types of vehicles involved, the number of casualties, their gender and severity of injuries, whether the police attended, and whether or not the crash victim was taken to hospital. No personal identifiers were recorded. The definition of a fatality was “a person who died on the spot of the crash site as a result of a road crash.” Injuries were categorized as severe if the injured party was hospitalized, and minor otherwise. There was no lower limit for property damage only; all were recorded. Monthly contact with the research team enabled review and retrieval of data, and clarification of queries.

#### Police records

De-identified individual crash data were obtained from routinely collected paper-based records held in the District Traffic Police Office. Two data collectors were trained to extract data onto paper data collection forms (Appendix Figure A6, online supplement), later entered into an electronic database.

#### Traffic data

Manual classified 24-hour traffic volume and pedestrian counts were conducted at two sites within each of the three lengths of highway using trained enumerators during July 2019. Pedestrians were counted moving along the road and crossing the road within 50 to 80 meters of the count location during a 16-hour time period. The counting sites were selected based on the presence of minor access roads, markets, and educational institutions nearby. Spot speed surveys in each direction of travel in peak and off-peak periods (8 am to 10 am; 12 pm to 2 pm; 4 pm to 6 pm; 8:30 pm to 10:30 pm) were conducted at two sites in each length of highway using a hand-held radar gun in October 2019.

### Data analysis

**Figure 1.**
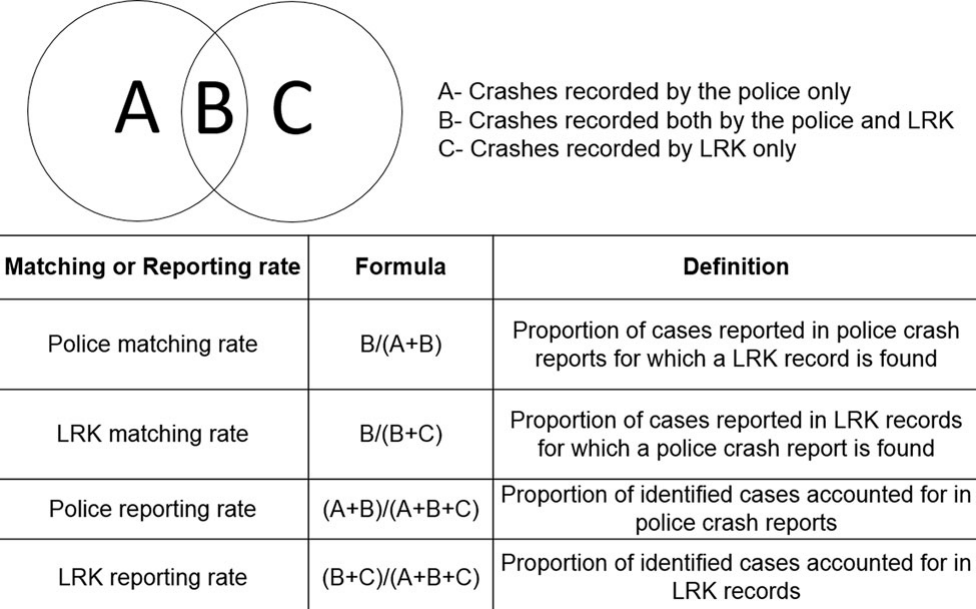
Definitions of reporting level (adapted from Doggett et al. 2018).

Analysis was carried out using SPSS 19.0. Police and LRK crash and casualty data were matched by date, time, and location based on locality descriptor, ward number, and point location (i.e., a landmark, e.g., a ward office, petrol pump, school, etc.). The reporting rate of the crashes and the matching rate between LRK and police data were computed according to Doggett et al. (2018), as shown in Figure 1. Differences in crash reporting by severity and vehicle type were explored inferentially using chi-square statistics. The odds ratio was used to measure an association between injury severity levels and data matching between LRK and police records. The variable “minor injury/property damage only” was used as the reference group.

### Ethical approval

Ethical approval was obtained from the Institutional Review Committee of Kathmandu Medical College and from the Faculty of Health and Applied Sciences Research Ethics Committee of the University of West of England, Bristol. A permission letter was obtained from Hetauda Municipality, Makwanpur.

## Results

We first present a short description of the traffic data. We then present the LRK and police data and make comparisons between the data. Finally, we assess data quality.

### Traffic data

The average daily traffic at Pashupatinagar, Nawalpur, and Basamadi was 18,707 vehicles/day, 14,056 vehicles/day, and 11,125 vehicles/day respectively. These are high flows. The flow on the access road to the industrial district at Pashupatinagar was 5,015 vehicles/day. The variations in traffic flow are likely to be related to the location proximity to Hetauda.

Almost two-thirds of the traffic comprised of motorized two-wheelers and trucks (Appendix Table A1, online supplement). Cars, jeeps, buses, utility vehicles, and motorized 2-wheelers have 85%ile speeds often near or exceeding the speed limit of 40 km/hr, especially at Basamadi and this results from the straight alignment of the road and absence of junctions (Appendix Table A2, online supplement). The difference in mean speed between trucks and the majority of other vehicle types is in the range 1–9 km/hr. The difference between the mean speed of trucks and the 85th percentile speed of faster types of vehicles is in the range 12–19 km/hr. Such differences in speed between vehicle types, coupled with their proportions in the traffic mix, are obvious possible contributors to crash risk. The pedestrian survey shows relatively high flows, with between 154 and 340 pedestrians/hour in the early evening period.

### Crash and injury data

As shown in Table 1, of the total of 110 crashes recorded by the LRKs, 70 were injury-related crashes. The total number of police-recorded crashes over the same period matched on crash details was 23. This number included 18 injury-related crashes.

**Table 1.** Comparison of LRK and police crash and injury data.

	Crashes/injuries recorded by the LRKs Number (percentage)	Crashes/injuries recorded by LRK as attended by the police Number (percentage of total recorded by LRKs)	Crashes/injuries recorded by the police Number (percentage)
Crashes with injuries	70 (63.6%)	35 (50.0%)	18 (78.3%)
Crashes without injuries	40 (36.4%)	11 (27.5%)	5 (21.7%)
Total number of crashes	110 (100.0%)	46 (41.8%)	23 (100.0%)
Fatality	5 (3.4%)	5 (100.0%)	8 (23.5%)
Severe injury	62 (42.2%)	45 (72.6%)	13 (38.2%)
Minor injury	78 (53.1%)	50 (64.1%)	6 (17.6%)
Unknown injury	2 (1.4%)	1 (50.0%)	7 (20.6%)
Total number of injuries	147 (100.0%)	101 (68.7%)	34 (100.0%)

The crashes recorded by the LRKs resulted in 5 fatalities, 62 severe injuries, 78 minor injuries, and 2 injuries of unknown severity. In the police record, 8 involved fatalities, 13 serious injuries, 6 minor injuries, and 7 injuries of unknown severity. The police visited all fatal crashes recorded by LRKs, 72.6% of severe injury crashes, 64.1% of minor injury crashes, and 27.5% of property damage only crashes. This bias to recording more fully the number of fatalities and higher injury severity by the police is in line with evidence from the police data alone, which was found to have 23.5% of records involving a fatality, and 38.2% involving a severe injury.

Both the LRK and police data reveal that males (76.9% and 61.8%, respectively) have higher crash involvement (Appendix Table A3, online supplement) than females. Fourteen of the 147 casualties (10%) were pedestrians, with one fatality and eight seriously injured. Fifty-eight of the 147 casualties were on motorized 2-wheelers (rider and pillion passenger), with three fatalities and 26 seriously injured.

Trucks (23.8% in the LRK data and 31.6% in the police data) and motorcyclists (41.1% and 26.3%) were associated with high crash involvement (Appendix Table A4, online supplement). Crashes involving trucks were associated with the highest police attendance (61.4% of the LRK recorded crashes involving trucks were attended by the police). This is in line with the police data which shows that the largest vehicle involvement type was trucks (31.6%). Vehicle with vehicle crashes were the most common (LRK data, 53.6%; Police data, 52.2%), followed by those involving pedestrians (12.7% and 34.8%). The higher proportion in the Police data indicates under-reporting of other types of crashes by the Police. The police did not attend single-vehicle crashes. The rate of police attendance at crashes was highest in Basamadi (54.5%), followed by Nawalpur (43.9%) and then Pashupatinagar (34%).

Police crash attendance at both Pashupatinagar and Nawalpur increased with the increase in injury severity level (Appendix Table A5, online supplement). However, police attendance at a bus crash involving 27 people with minor injuries skewed the data in Basamadi.

#### Quality of the data

**Figure 2.**
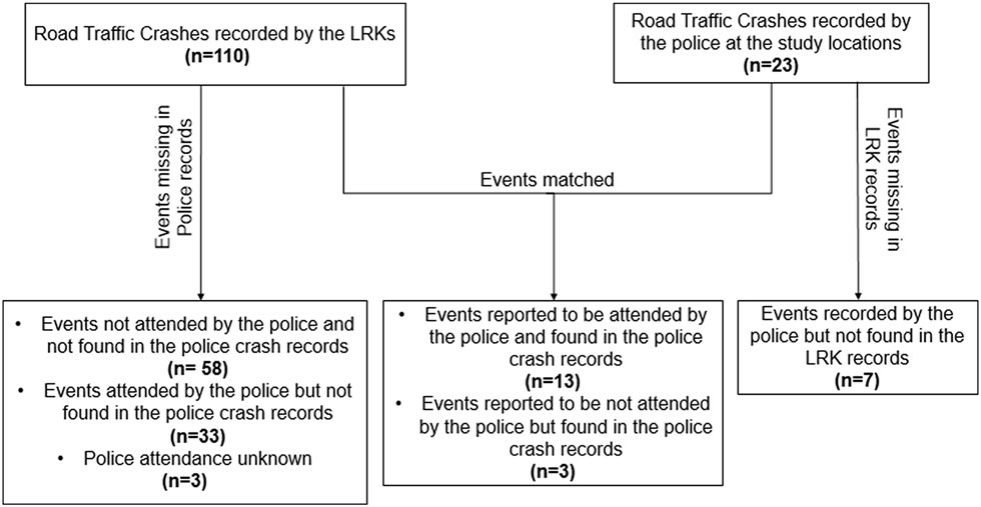
Matched/missing records between LRK and police data.

The LRKs were diligent in crash reporting with ten of the fifteen data fields in the reporting forms 100% complete, and the remaining five fields being between 97.3% and 98.6% complete. Figure 2 summarizes the matching of LRK and police data. Of 110 crashes reported by the LRKs, 46 (41.8%) were recorded by the LRKs as having been attended by the police. However, only 16 crashes appear in both the LRK and police data. Of these 16 crashes, it should be noted that for three of them, the LRK records suggest that the police did not attend. For a further four of these 16, the date and time did not match, though details such as vehicle type, vehicle direction of travel, crash type and injury outcome, indicated they were the same crash. Either the police or the LRK date and time were inaccurate.

Table 2 below presents the reporting and matching rates for the police and LRK data.

**Table 2.** Reporting and matching rate for police and LRK data.

	Only in Police data	In Police and LRK data	Only in LRK data	Reporting rate		Matching rate	
				Police	LRK	Police	LRK
Description	A	B	C	(A + B)/(A + B + C)	(B + C)/(A + B + C)	B/(A + B)	B/(B + C)
Total crash events	7	16	94	19.7%	94.0%	69.6%	14.5%
Fatality	3	5	0	62.5%	62.5%	62.5%	100%
Severe injury	4	9	53	19.7%	93.9%	69.2%	14.5%
Minor injury	6	0	78	7.1%	92.9%	0.0%	0.0%
Injury-unknown	7	0	2	77.8%	22.2%	0.0%	0.0%
Damage only	3	2	38	11.6%	93.0%	40.0%	5.0%
Single-vehicle	0	1	25	3.8%	100%	100%	3.8%
Vehicle-Vulnerable road user	2	6	10	44.4%	88.9%	75.0%	37.5%
Vehicle-vehicle	4	9	59	18.1%	94.4%	69.2%	13.2%

The police reporting rate is 19.7% (23 of 117, 95% CI 13.4 to 27.7), while the LRK reporting rate is 94.0% (110 of 117, 95% CI 88.1 to 97.0). The police reporting rate is highest for fatalities (62.5%) but only 19.7% for severe injuries and 7.1% for minor injuries. Except for fatalities, the LRK reporting is higher than the police rate, at 93.9% for serious injuries and 92.9% for minor injuries. Police reporting rates are significantly lower than LRK reporting rates, especially for single vehicle and vehicle-to-vehicle crashes. The rate for crashes involving vulnerable road uses is higher, but still only half that of the LRK data.

The LRK matching rate is 14.5% and indicates the low proportion of crashes in LRK data that are also reported in the police data. The LRK matching rate for fatality was 62.5% (5/8); which is probably due to differences in the definition of fatality used by the police and the LRKs. The police matching rate for vulnerable road users is higher than for other classes of crash.

There is a significant association between injury severity in a crash (fatality and serious injury versus minor injury and damage only) and whether the crash is recorded in both the police and LRK data (χ^2^(1) = 19.94, p < 0.001). Crashes with more severe injuries are 15.3 times more likely to be reported in police data as well as LRK data. No significant association was found between the type of crash (multiple vehicles versus single-vehicle) and whether the crash is recorded in both the police and LRK data (χ^2^(1) = 1.74, p = 0.187).

## Discussion

The study applied a methodology employing shopkeepers as road crash Local Record Keepers, and compared the quality of their data with police data. The findings illustrate that at these locations police data capture only a small proportion of total crashes. As compared to the reporting rate of 94% in LRK data, the reporting rate of the police was only 19.6%. Our approach indicated similar levels of under-reporting in police data, as seen in hospital linkage studies. The under-reporting rate of 80.4% was similar to that reported by Singh et al. (2018) (85%) in Chandigarh, India comparing police and hospital data. Periyasamy et al. (2013) used community-based cross-sectional surveys in Kandy, Sri Lanka to estimate police under-reporting and found it to be between 33 and 56%. Unlike hospital records and surveys that capture only crashes resulting in injury or fatality, the LRK records in this study comprise of property damage as well and therefore provide a more complete picture of under-reporting.

Studies have found the reporting rate in police data improves with increasing injury severity (Elvik and Mysen 1999; Salifu and Ackaah 2012). The reporting rate for the police in this study showed similar findings. Police reported 100% of fatal casualties reported by the LRKs suggesting the police data are reliable for fatal crashes. This might be because a police representative is assigned to each hospital in Nepal to record and report road crash fatalities for further investigation. Police data were found to be less complete for minor injuries and damage-only crashes. As in many countries, police categorization of minor and severe injury is limited by the lack of formal definitions and medical training in the police, resulting in decisions being based on personal opinion. Adopting a standard measure of injury severity such as the KABCO injury scale (Popkin et al. 1991) together with training, could improve injury severity assessment and reporting.

We found that police crash attendance and reporting varied by type of collision. This is consistent with Salifu and Ackaah (2012), who compared the police crash data in Ghana with data collected from surveys at hospitals and drivers. They found that the reporting was low for the single-vehicle collisions and ones that did not involve pedestrians.

Nepal police traffic crash records mostly focus on driver behaviors such as overloading, speeding, and inappropriate overtaking (Thapa 2013). They seldom report other factors, such as vehicle, road, and environmental factors that could contribute to crash risk. This may suggest that, where there is a victim, the police seek an offender.

A high proportion (71.7%) of crashes that the LRKs reported were attended by police were not found in the police records. Despite the police attending the crash sites and Motor Vehicle and Transport Management Act (1993) stating that drivers should report crashes, recording deficiencies and non-reporting are likely to happen where disputes over fault are resolved through a negotiated settlement. The barriers and facilitators to crash reporting to the police and by the police need to be further explored.

The key strength of this study is the direct involvement of members from the local community and this could encourage ownership of road safety-related activities, and ensure the sustainability of activities such as crash recording through the transfer of knowledge among community members. The simplicity in setting up and operating LRK methodology, led to rapid learning, and fast and successful implementation. Our selection and recruitment process resulted in the engagement of shopkeepers who were very interested in the problem of RTCs, resulting in strong commitment of the shopkeepers over the 12 month duration of the study and high levels of completeness of the data they collected. Besides, the method captured not only crashes resulting in a fatality or severe injury but also minor injury and property damage, which would generally go unreported.

One limitation of the study is that the definition of fatality and injury severities used by the LRKs does not correspond with the standard definitions used by Nepal police (such as a road traffic death being one occurring within 30 days of a crash). Like the police, the LRKs do not have medical training, therefore risking under or over-reporting of injury severities. In the absence of personal identifiers, and the police records lacking coordinates of the crash location, it may be difficult to ascertain whether a crash occurred within the study boundary. Inconsistencies in the date and time in a few of the matched records suggest that further work is needed to enhance the accuracy of reporting.

Traffic police in other districts of Nepal may differ in the proportion of crashes they report, perhaps due to variations in, for example, manpower, resources, training, and senior officer interest. However, the level of underreporting in police data found in our study in Makwanpur suggests that decision-makers should exercise caution when making policy, legislation, and strategy decisions based solely on these data. Similarly, those seeking to use traffic police data to monitor trends in crashes or to evaluate the impact of an intervention or policy change should be cautious, particularly when examining non-fatal crashes Estimation of underreporting levels in other areas, may find using methods as described in this study helpful.

The recent implementation of a web-based road accident information management system (RA-IMS) by the Department of Transportation Management in Nepal may improve crash reporting, however evidence from this study suggests that reporting may still be incomplete if crashes are not reported to the police, or the police do not routinely attend and/or record crashes. Issues with the management of the tasks of the police persist in Nepal, with limited numbers of police having a large inventory of duties and responsibilities (e.g., managing traffic, facilitating the movement of VIPs, etc.). Training, logistical support, and adequate human resources will need to be provided. Road users need to be encouraged to report all road crashes by easing the legal procedures and reducing the time commitment required for reporting. In the absence of other alternative data sources, the LRK methodology is feasible and effective for ascertaining the quality of police crash data and advocating for improvements in crash data collection and reporting.

## Supplementary Material

Supplemental Material

## Data Availability

The data that support the findings of this study are available from the corresponding author (AK), upon request.
