# Supplementary material for: Completeness of police reporting of traffic crashes in Nepal: Evaluation using a community crash recording system
Source: Traffic Inj Prev. 2022 Jan 14;23(2):79–84. doi: 10.1080/15389588.2021.2012766 (PMC8862739; doi:10.1080/15389588.2021.2012766)
Supplement: Supplemental Material [file GCPI_A_2012766_SM8789.zip › Appendix.docx]

**APPENDICES**

**Bibliography**

Magnus D, Bhatta S, Mytton J, Joshi E, Bird EL, Bhatta S, Manandhar SR, Joshi SK. 2020. Establishing injury surveillance in emergency departments in Nepal: protocol for mixed methods prospective study. BMC Health Serv. Res. 20(1):433. doi:10.1186/s12913-020-05280-9.

Rosman DL. 2001. The Western Australian Road Injury Database (1987–1996):: Ten years of linked police, hospital and death records of road crashes and injuries. Accid Anal Prev*.* 33(1):81-88. doi:10.1016/S0001-4575(00)00018-X.

Van HT, Singhasivanon P, Kaewkungwal J, Suriyawongpaisal P, Khai LH. 2006. Estimation of non-fatal road traffic injuries in Thai Nguyen, Vietnam using capture-recapture method. Southeast Asian J Trop Med Public Health. 37(2):405-411. PMID:17125007.

World Health Organization, 2019. Global status report on road safety 2019. World Health Organization.

**Figure A1: Study location, Makwanpur, Nepal (Source: Map data: Google, Maxar Technologies, CNES/Airbus, 2020).**


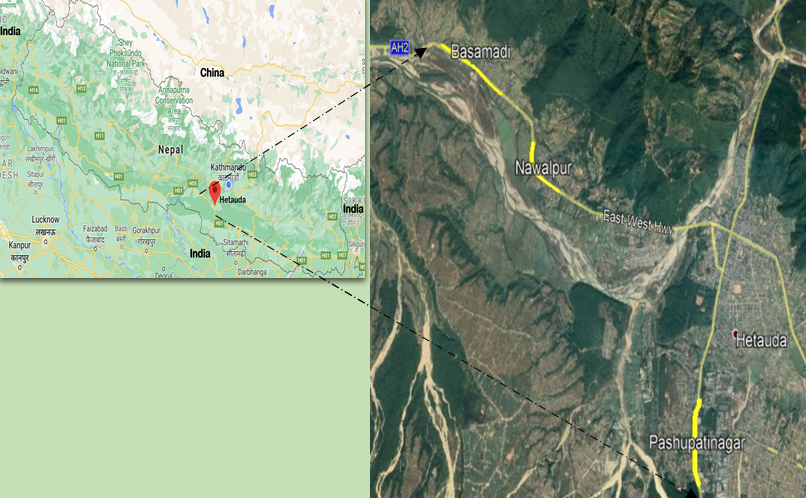


Note: “Yellow= Study locations”

**Figure A2: Pashupatinagar road scenes and boundaries (Police, red; LRK yellow) (Source: Map data: Google, Maxar Technologies, CNES/Airbus, 2020).**


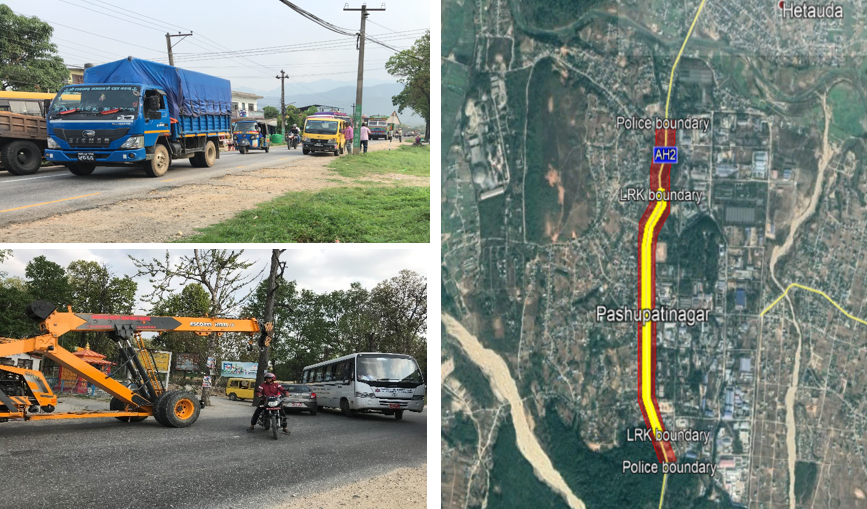


Note: “Red= Police reporting boundary, Yellow= LRK reporting boundary”

**Figure A3: Nawalpur road scenes and boundaries (Police, red; LRK yellow) (Source: Map data: Google, Maxar Technologies, CNES/Airbus, 2020).**


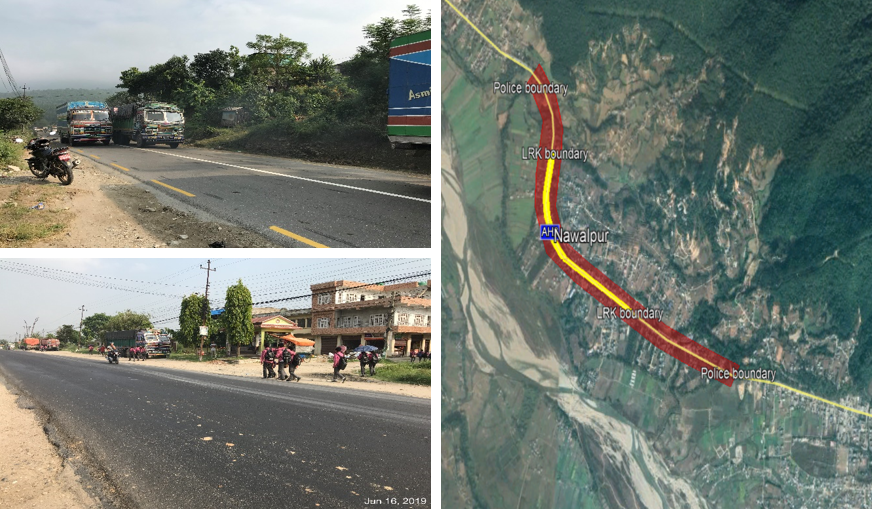


Note: “Red= Police reporting boundary, Yellow= LRK reporting boundary”

**Figure A4: Basamadi road scenes and boundaries (Police, red; LRK yellow) (Source: Map data: Google, Maxar Technologies, CNES/Airbus, 2020).**


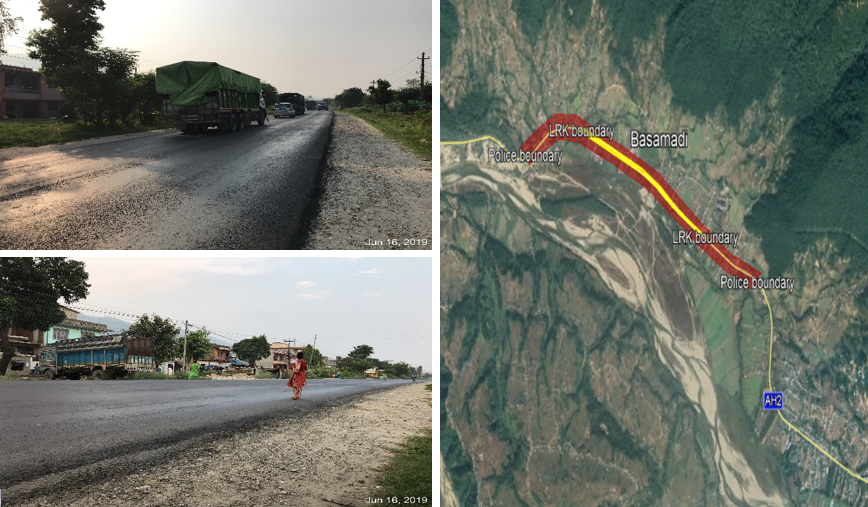


Note: “Red= Police reporting boundary, Yellow= LRK reporting boundary”

**Figure A5: Local Record Keeper data collection form.**

**
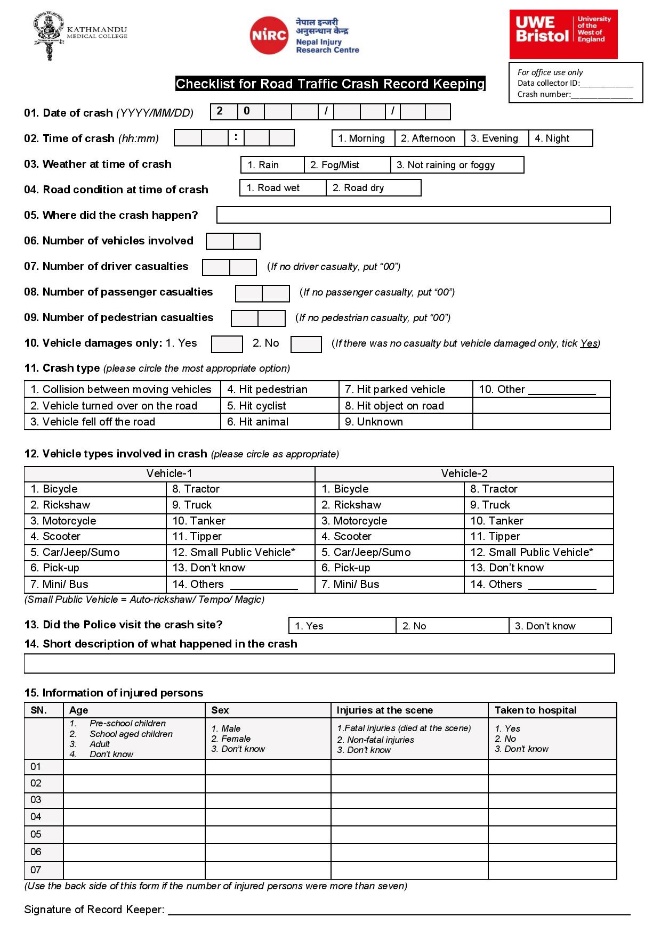
**

**Figure A6: Police data collection form.**

**Page 1**

**
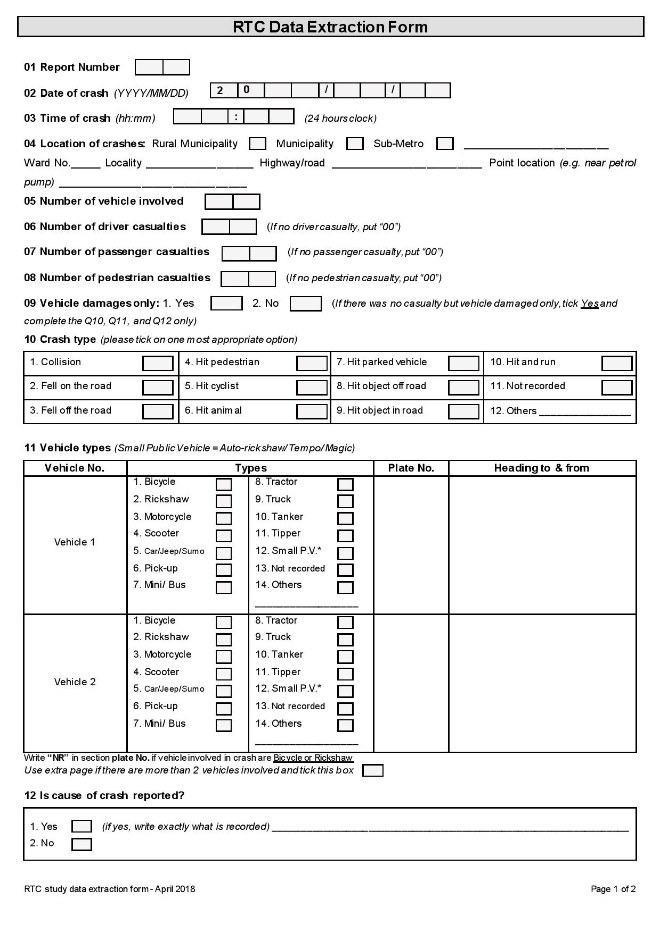
**

**Figure A7: Police data collection form (Continued).**

**Page 2**

**
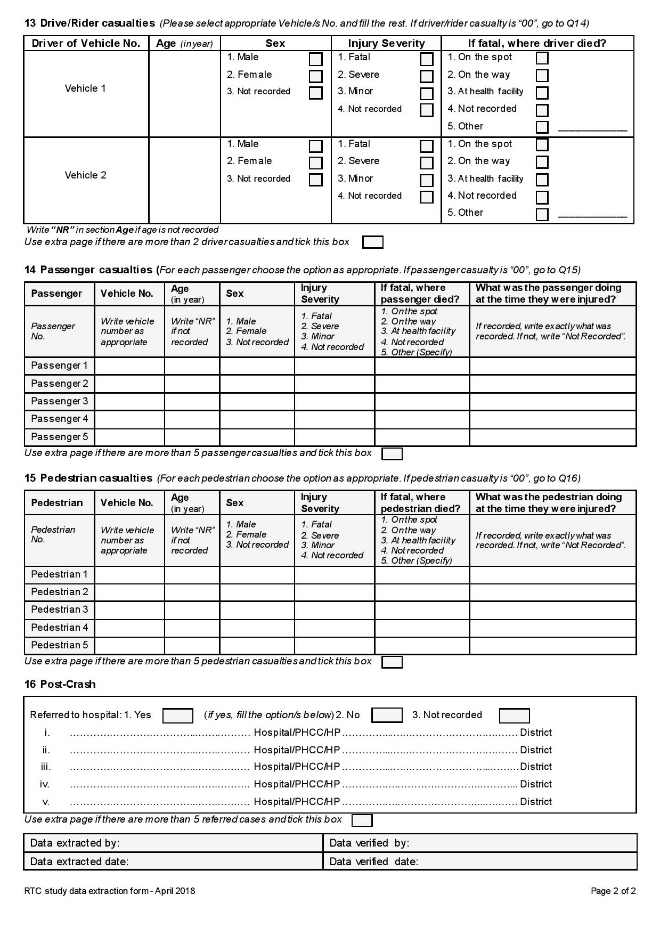
**

**Table A1. Vehicle type in the flow of traffic (percentages).**

|  | **Pashupatinagar** | **Nawalpur** | **Basamadi** |
| --- | --- | --- | --- |
| Motorized 2-wheelers | 36 | 33 | 29 |
| Trucks | 25 | 29 | 34 |
| Cars / Jeeps | 14 | 13 | 15 |
| Motorized 3-wheelers | 9 | 9 | 5 |
| Buses | 5 | 5 | 5 |
| Utility vehicle | 4 | 4 | 4 |
| Tractor | 1 | 1 | 1 |
| Other | 6 | 6 | 7 |
| Total | 100 | 100 | 100 |

**Table A2. Vehicle speed details (km/hr).**

|  | **Pashupatinagar** | | **Nawalpur** | | **Basamadi** | |
| --- | --- | --- | --- | --- | --- | --- |
|  | **Mean (Standard deviation)** | **85^th^ %ile** | **Mean (Standard deviation)** | **85th %ile** | **Mean (Standard deviation)** | **85th %ile** |
| Car | 31 (10) | 41 | 27 (10) | 38 | 32 (11) | 44 |
| Jeep | 34 (11) | 45 | 32 (11) | 43 | 40 (10) | 50 |
| Bus | 29 (9) | 39 | 29 (9) | 38 | 35 (9) | 44 |
| Utility vehicle | 31 (9) | 41 | 28 (11) | 39 | 34 (10) | 44 |
| Motorized 2-wheeler | 31 (9) | 40 | 28 (9) | 38 | 34 (10) | 45 |
| Motorized 3-wheeler | 25 (5) | 31 | 22 (5) | 28 | 28 (6) | 34 |
| Truck | 27 (6) | 34 | 25 (7) | 33 | 31 (6) | 38 |
| Tractor | 20 (4) | 25 | 17 (3) | 20 | 18 (3) | 21 |

**Table A3. Involvement by gender and injury types for pedestrians and motorized 2-wheelers.**

|  | **Recorded by the LRKs**  **Number (percentage)** | **Attended by the police**  **Number (percentage of total recorded by LRKs)** | **Recorded by the police**  **Number (percentage)** |
| --- | --- | --- | --- |
| **Involvement by gender** | | | |
| Male | 113 (76.9%) | 73 (64.6%) | 21 (61.8%) |
| Female | 31 (21.1%) | 25 (80.6%) | 7 (20.6%) |
| Unknown | 3 (2.0%) | 3 (100%) | 6 (17.6%) |
| **Total number of injuries** | **147 (100.0%)** | **101 (68.7%)** | **34 (100.0%)** |
| **Injury type** | | | |
| Pedestrian fatality | 1 (7.1%) | 1 (100.0%) | 3 (37.5%) |
| Pedestrian severe injury | 8 (57.1%) | 6 (75.0%) | 3 (37.5%) |
| Pedestrian minor injury | 5 (35.7%) | 0 (0.0%) | 1 (12.5%) |
| Pedestrian unknown injury | 0 (0.0%) | 0 (0.0%) | 1 (12.5%) |
| **Pedestrians total** | **14 (100.0%)** | **7 (50.0%)** | **8 (100.0%)** |
| Motorized 2-wheeler user fatality | 3 (5.2%) | 3 (100.0%) | 3 (50.0%) |
| Motorized 2-wheeler user severe injury | 26 (44.8%) | 16 (61.5%) | 1 (16.7%) |
| Motorized 2-wheeler user minor injury | 29 (50.0%) | 10 (34.5%) | 0 (0.0%) |
| Motorized 2-wheeler user unknown injury | 0 (0.0%) | 0 (0.0%) | 2 (33.3%) |
| **Motorized 2-wheeler user total** | **58 (100.0%)** | **29 (50.0%)** | **6 (100.0%)** |

**Table A4. Involvement by vehicle type, and type of interaction.**

|  | **Recorded by the LRKs**  **Number (percentage)** | **Attended by the police**  **Number (percentage of total recorded by LRKs)** | **Recorded by the police**  **Number (percentage)** |
| --- | --- | --- | --- |
| **Involvement by type of vehicle** | | | |
| Motorized 2-wheelers | 76 (41.1%) | 27 (35.5%) | 10 (26.3%) |
| Trucks | 44 (23.8%) | 27 (61.4%) | 12 (31.6%) |
| Cars/Jeeps | 23 (12.4%) | 11 (47.8%) | 7 (18.4%) |
| Motorized 3-wheelers and ‘Magics’ | 18 (9.7%) | 10 (55.6%) | 1 (2.6%) |
| Buses | 8 (4.3%) | 4 (50.0%) | 3 (7.9%) |
| Pick-up | 6 (3.2%) | 3 (50.0%) | 2 (5.3%) |
| Tipper | 5 (2.7%) | 2 (40.0%) | 2 (5.3%) |
| Trailer | 2 (1.1%) | 2 (100.0%) | 0 (0.0%) |
| Bicycle | 2 (1.1%) | 0 (0.0%) | 0 (0.0%) |
| Tractor | 1 (0.5%) | 0 (0.0%) | 1 (2.6%) |
| **Vehicle types total** | **185 (100.0%)** | **86 (46.5%)** | **38 (100.0%)** |
| **Type of interaction** | | | |
| Vehicle-Vehicle | 59 (53.6%) | 31 (52.5%) | 12 (52.2%) |
| Vehicle-Parked Vehicle | 9 (8.2%) | 4 (44.4%) | 1 (4.3%) |
| Vehicle-Pedestrian | 14 (12.7%) | 7 (50.0%) | 8 (34.8%) |
| Vehicle-Cyclist | 2 (1.8%) | 0 (0.0%) | 0 (0.0%) |
| Vehicle-Animal | 6 (5.5%) | 0 (0.0%) | 1 (4.3%) |
| Single Vehicle (rollover) | 12 (10.9%) | 0 (0.0%) | 0 (0.0%) |
| Single Vehicle (left carriageway) | 8 (7.3%) | 4 (50.0%) | 0 (0.0%) |
| Other | 0 (0.0%) | 0 (0.0%) | 1 (4.3%) |
| **Crash types total** | **110 (100.0%)** | **46 (41.8%)** | **23 (100.0%)** |

**Table A5. LRK data by location.**

|  | **Pashupatinagar** | | **Nawalpur** | | **Basamadi** | |
| --- | --- | --- | --- | --- | --- | --- |
|  | **Crash outcome**  **Number / (percentage of total)** | **Of which the Police attended**  **Number / (percentage attended)** | **Crash outcome (total)**  **Number / (percentage of total)** | **Of which the Police attended**  **Number / (percentage attended)** | **Crash outcome (total)**  **Number / (percentage of total)** | **Of which the Police attended**  **Number / (percentage attended)** |
| Crash with injury | 27 (57.4%) | 12 (44.4%) | 31 (75.6%) | 15 (48.4%) | 12 (54.5%) | 8 (66.7%) |
| Crash with property damage only | 20 (42.6%) | 4 (20.0%) | 10 (24.4%) | 3 (30.0%) | 10 (45.5%) | 4 (40.0%) |
| Crash Total | 47 (100.0%) | 16 (34.0%) | 41 (100.0%) | 18 (43.9%) | 22 (100.0%) | 12 (54.5%) |
| Fatal | 0 (0.0%) | 0 (0.0%) | 1 (2.0%) | 1 (100.0%) | 4 (8.7%) | 4 (100.0%) |
| Severe injury | 29 (58.0%) | 23 (79.3%) | 24 (47.1%) | 16 (66.7%) | 9 (19.6%) | 6 (66.7%) |
| Minor injury | 20 (40.0%) | 6 (30.0%) | 26 (51.0%) | 14 (53.8%) | 32 (69.6%) | 30 (93.8%) |
| Unknown injury | 1 (2.0%) | 0 (0.0%) | 0 (0.0%) | 0 (0.0%) | 1 (2.2%) | 1 (100.0%) |
| Total | 50 (100.0%) | 29 (58.0%) | 51 (100.0%) | 31 (60.8%) | 46 (100.0%) | 41 (89.1%) |
