# Supplementary figures and images for: Completeness of police reporting of traffic crashes in Nepal: Evaluation using a community crash recording system
Source: Traffic Inj Prev. 2022 Jan 14;23(2):79–84. doi: 10.1080/15389588.2021.2012766 (PMC8862739; doi:10.1080/15389588.2021.2012766)

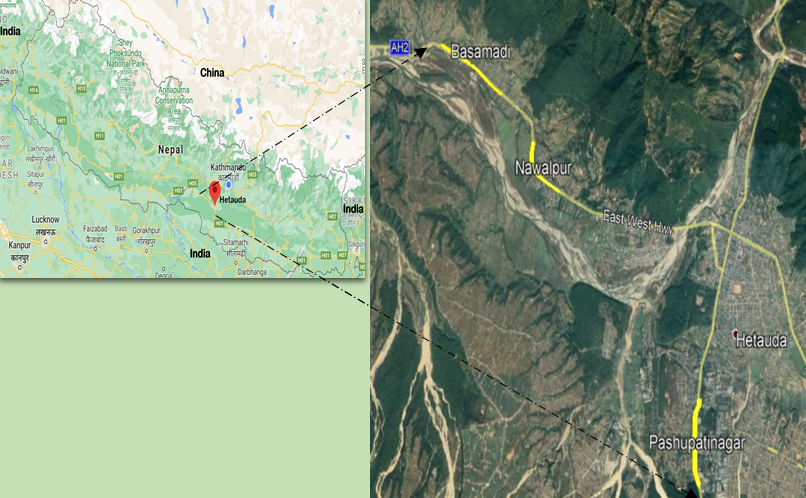

Supplement: Supplemental Material [file GCPI_A_2012766_SM8789.zip › Figure A1.png]

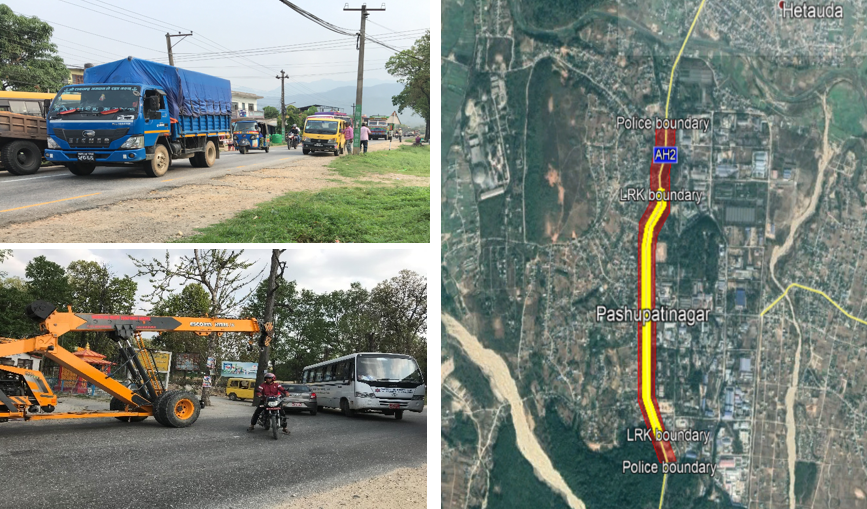

Supplement: Supplemental Material [file GCPI_A_2012766_SM8789.zip › Figure A2.png]

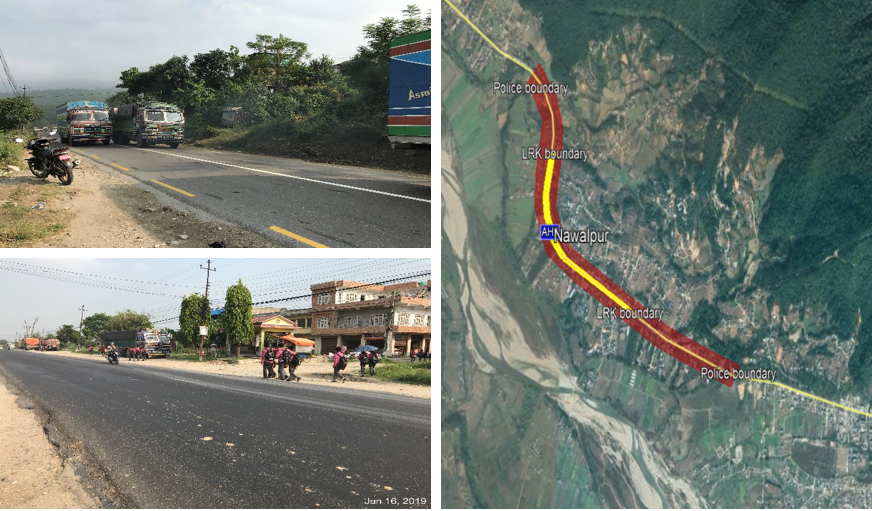

Supplement: Supplemental Material [file GCPI_A_2012766_SM8789.zip › Figure A3.png]

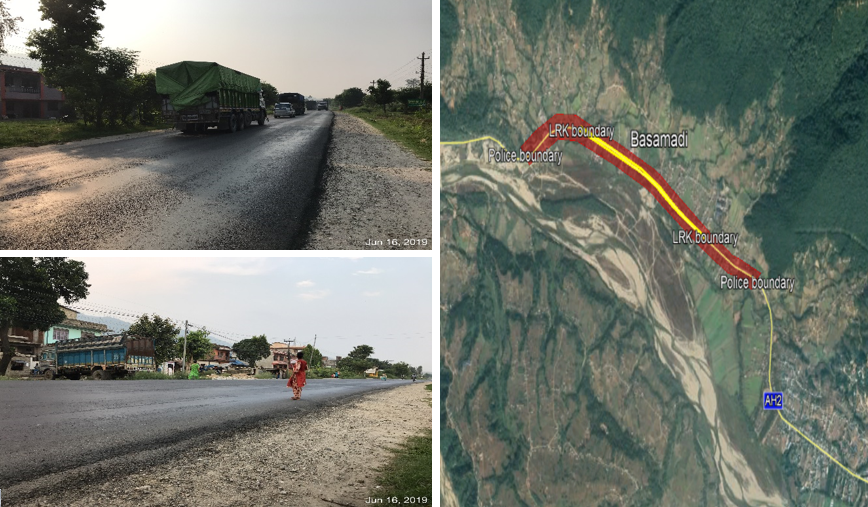

Supplement: Supplemental Material [file GCPI_A_2012766_SM8789.zip › Figure A4.png]

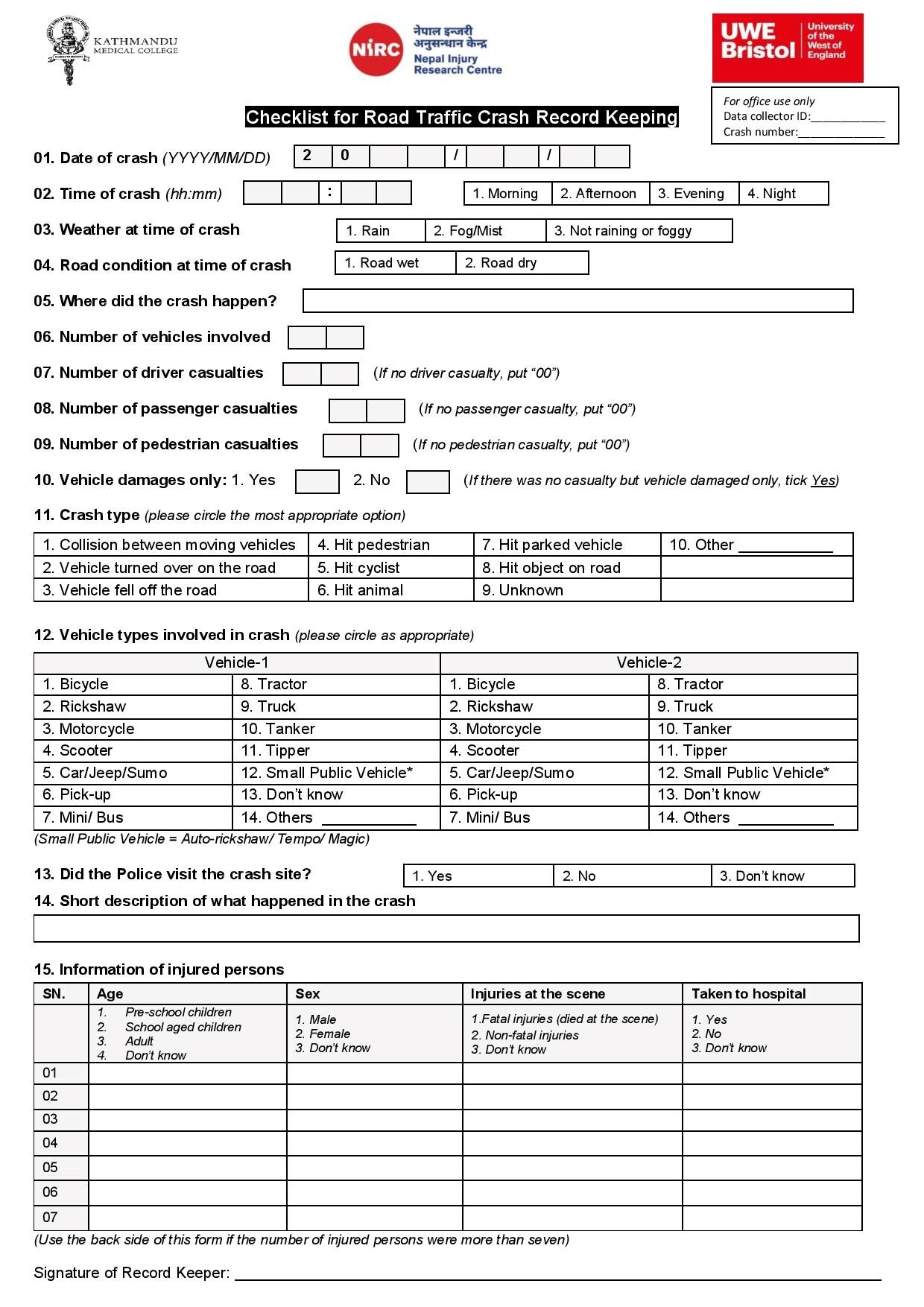

Supplement: Supplemental Material [file GCPI_A_2012766_SM8789.zip › Figure A5.jpg]

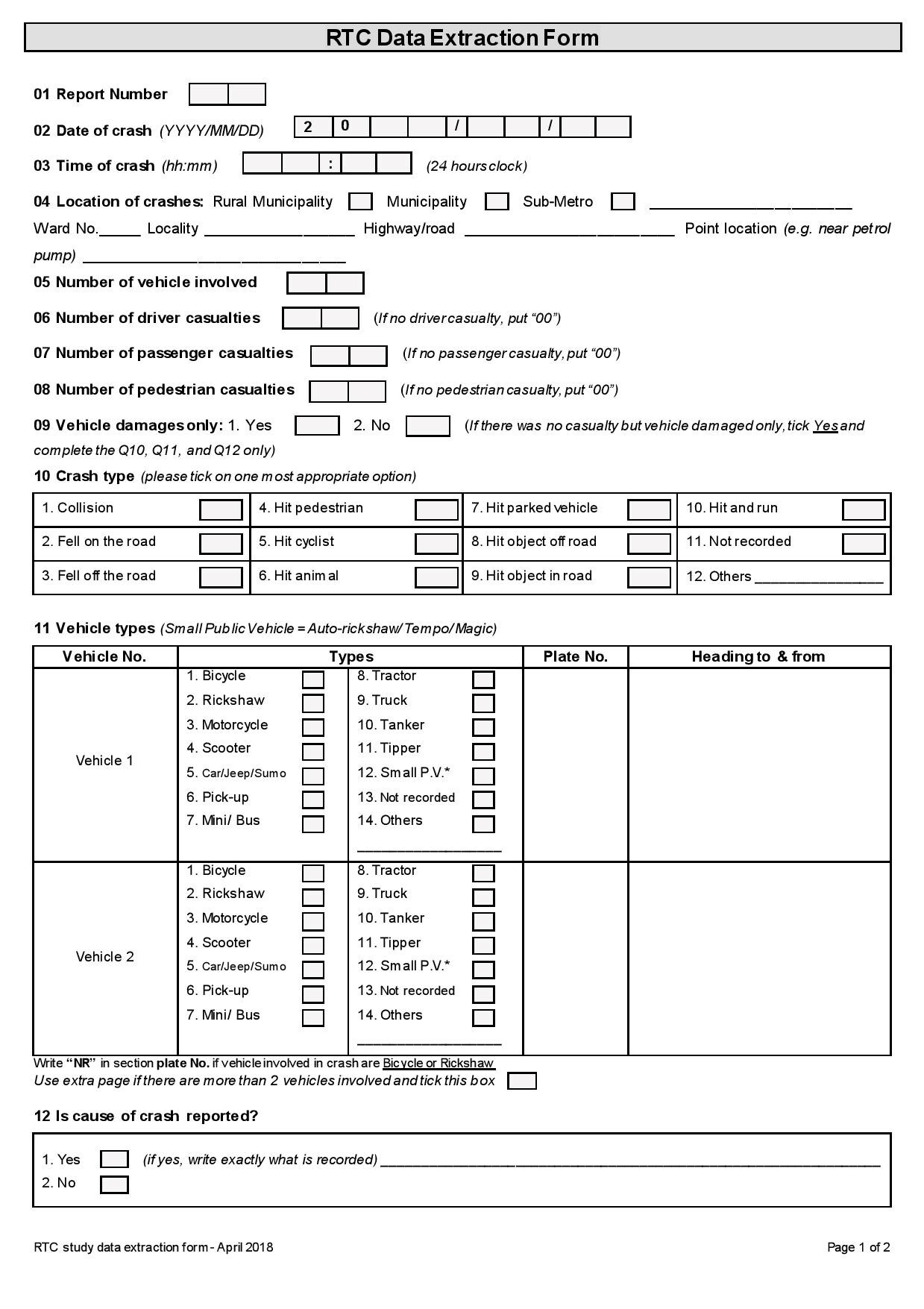

Supplement: Supplemental Material [file GCPI_A_2012766_SM8789.zip › Figure A6.jpg]

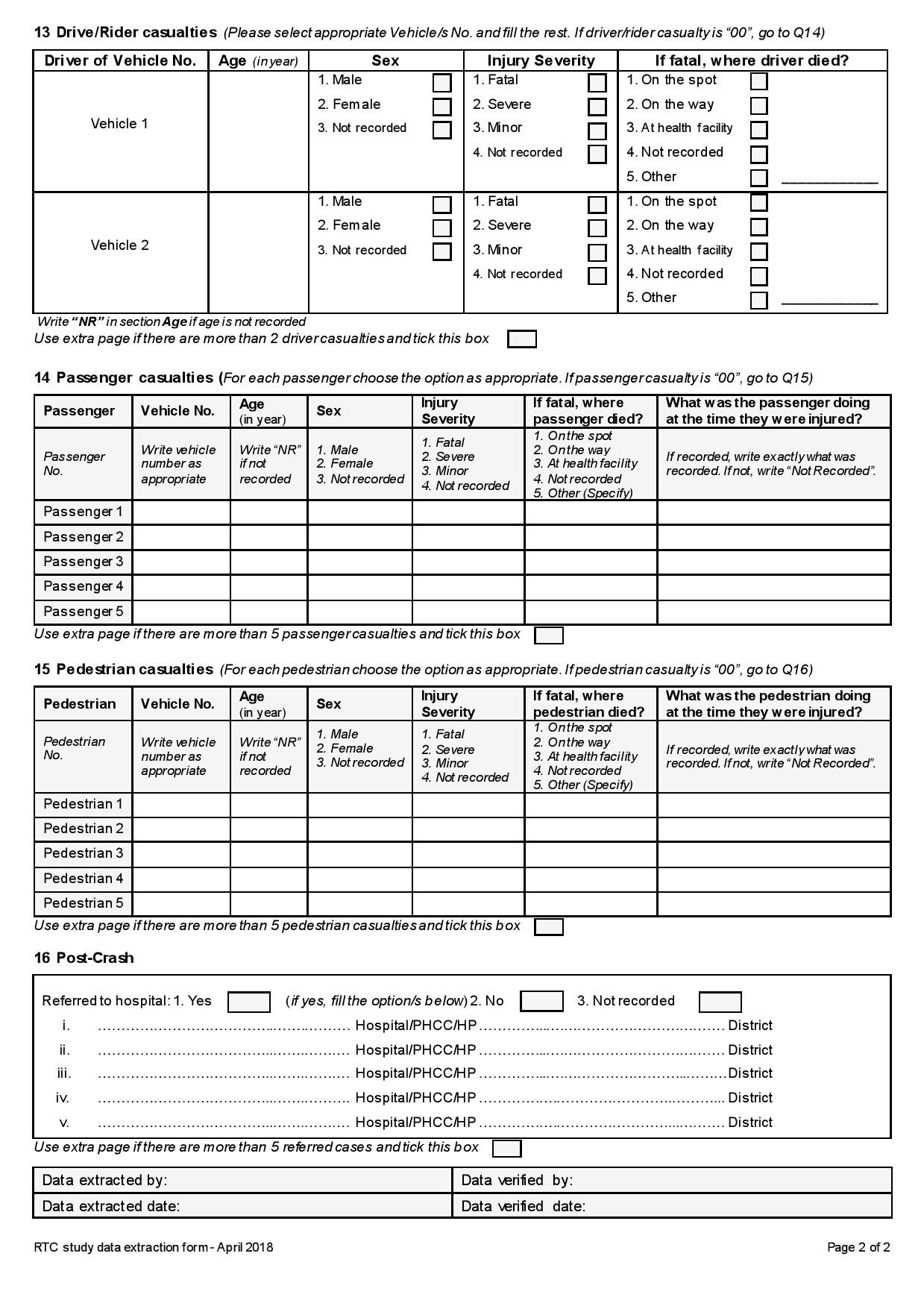

Supplement: Supplemental Material [file GCPI_A_2012766_SM8789.zip › Figure A7.jpg]
